# Sex Pheromone Receptor Ste2 Orchestrates Chemotropic Growth towards Pine Root Extracts in the Pitch Canker Pathogen *Fusarium circinatum*

**DOI:** 10.3390/pathogens13050425

**Published:** 2024-05-17

**Authors:** Jane B. Ramaswe, Emma T. Steenkamp, Lieschen De Vos, Felix F. Fru, Omotayo O. Adegeye, Brenda D. Wingfield

**Affiliations:** Department of Biochemistry, Genetics and Microbiology, Forestry and Agricultural Biotechnology Institute (FABI), University of Pretoria, Pretoria 0002, South Africa; jane.ramaswe@fabi.up.ac.za (J.B.R.); lieschen.bahlmann@fabi.up.ac.za (L.D.V.); felixfr30@gmail.com (F.F.F.); tayoadenig@gmail.com (O.O.A.); brenda.wingfield@fabi.up.ac.za (B.D.W.)

**Keywords:** chemotropism, split-marker constructs, G protein-coupled receptors (GPCRs), peptide pheromone, heterothallism, autocrine signaling, paracrine signaling

## Abstract

In ascomycetous fungi, sexual mate recognition requires interaction of the Ste2 receptor protein produced by one partner with the α-factor peptide pheromone produced by the other partner. In some fungi, Ste2 is further needed for chemotropism towards plant roots to allow for subsequent infection and colonization. Here, we investigated whether this is also true for the pine pitch canker fungus, *Fusarium circinatum*, which is a devastating pathogen of pine globally. Ste2 knockout mutants were generated for two opposite mating-type isolates, after which all strains were subjected to chemotropism assays involving exudates from pine seedling roots and synthetic α-factor pheromone, as well as a range of other compounds for comparison. Our data show that Ste2 is not required for chemotropism towards any of these other compounds, but, in both wild-type strains, Ste2 deletion resulted in the loss of chemotropism towards pine root exudate. Also, irrespective of mating type, both wild-type strains displayed positive chemotropism towards α-factor pheromone, which was substantially reduced in the deletion mutants and not the complementation mutants. Taken together, these findings suggest that Ste2 likely has a key role during the infection of pine roots in production nurseries. Our study also provides a strong foundation for exploring the role of self-produced and mate-produced α-factor pheromone in the growth and overall biology of the pitch canker pathogen.

## 1. Introduction

Sensing and responding to environmental cues are vital for all living organisms. This is particularly true for soil-dwelling microorganisms that use complex, coordinated processes to communicate with their surroundings [1,2,3]. These organisms can sense a variety of chemical signals, and many can then respond by manipulating the surroundings for their benefit [4,5]. For example, filamentous fungi can sense chemical stimuli and direct their growth toward or away from such compounds [6]. The directed growth in response to chemical gradients is broadly referred to as chemotropism, which is a well-known process in many fungi, as it promotes access to nutrients and contact with mating partners [7,8].

Research conducted during the last decade has demonstrated that chemotropism plays a vital role in the pathogenesis of certain fungal pathogens of plants [9,10]. This is because, in addition to mediating the directed growth of soil-inhabiting pathogens towards or away from certain chemicals [11,12], chemotropism also seems to afford fungi the ability to grow towards plant roots [4,13,14]. Previous studies have shown that chemotropism, involving chemical signals in root exudates from tomato and wheat, is involved in the colonization of these plants by *Fusarium oxysporum* and *Fusarium graminearum*, respectively [15,16,17]. However, it is not yet well understood if and how this process might affect root-colonization in other phytopathogens.

Mechanistically, the process underlying chemotropism towards plant roots in *F. oxysporum* and *F. graminearum* has been co-opted from their sexual mating systems [18]. In Ascomycota, the latter is governed by the so-called MAT1 locus, which in heterothallic fungi encodes distinct sequences for defining mating-type identity and for regulating the expression of corresponding mating peptide pheromones [19]. Accordingly, MAT1-2 cells usually express **a**-pheromone, while MAT1-1 cells express α-pheromone, thus leading to distinct expression levels of the corresponding pheromone receptors on the surface of opposite-mating-types cells [20]. Mating between the two partners is triggered when the α- and **a**-pheromones, respectively, interact with their cognate Guanine nucleotide-binding protein (G protein)-coupled receptors (GPCRs) Ste2 and Ste3 [19,21,22,23]. Homothallic Ascomycota also have the MAT1 locus, but their expression of mating pheromones is generally less stringently regulated. This is likely because the recognition of mating partners during recombination is not essential for selfing, as outcrossing is not obligatory for most homothallic fungi [19].

In their seminal work on *F. oxysporum*, Turrà and colleagues [15] demonstrated that this pathogen uses its α-pheromone receptor, Ste2, for chemotropic sensing of plant signals and ultimate colonization of tomato roots. The response appears to be triggered by class III peroxidases released by the roots and transduced by the cell wall integrity (CWI) mitogen-activated protein kinase (MAPK) pathway [10,15] and the NADPH oxidase complex [14,24,25]. The fungus also exhibits positive chemotropic behavior in response to synthetic α-peptide pheromone, which is unexpected for an asexual fungus [15,23]. Although *F. oxysporum* is thought to have heterothallic origins [26,27], evidence of sexual reproduction is lacking [23]. Nevertheless, for the homothallic pathogen, *F. graminearum*, Sridhar and colleagues [17] reported similar findings and showed that it also exhibited Ste2-mediated positive chemotropism towards synthetic α-peptide pheromone, as well as towards root exudates of its host plant. No equivalent study has been conducted on a bona fide heterothallic member of *Fusarium.*

Here, we investigated whether Ste2-mediated chemotropism could play a role in the pathogenesis of *F. circinatum*. This highly invasive and devastating pathogen of pine is best known for the pitch-soaked cankers it causes on susceptible pine species occurring in plantations and natural stands [28]. However, the pathogen can also infect the roots of pine seedlings, resulting in severe root and root collar disease [28]. Previous work has shown that hyphae of *F. circinatum* require natural openings or wounds through which to penetrate roots [29], thus suggesting the ability to sense chemical signals released by roots and to direct hyphal growth towards the plant [9,29]. Furthermore, in terms of sexual reproduction, *F. circinatum* is heterothallic with both mating types commonly encountered in natural populations [30,31] and the fungus readily undergoes sexual reproduction when fertile isolates of opposite mating type interact [32]. Therefore, in contrast to *F. graminearum* and *F. oxysporum*, sexual reproduction in *F. circinatum* is governed by a typical heterothallic system in which the two forms of the MAT1 locus (i.e., MAT1-1 and MAT1-2) dictate expression of the α- and **a**-peptide pheromones, as well as the Ste2 and Ste3 pheromone receptors [30].

The overall aim of this study was to characterize the chemotropic behavior potentially mediated by the Ste2 sex pheromone receptor in *F. circinatum*. For this purpose, we generated ∆*ste2* deletion mutants for field-collected MAT1-1 and MAT1-2 isolates of *F. circinatum.* Their chemotropic responses were then examined in the presence of various chemical compounds, including synthetic α-pheromone peptide and pine seedling root exudate. The study would thus contribute to our overall understanding of the infection biology of *F. circinatum*, as the root disease it causes in nursery seedlings often represents a significant hurdle to pine-based forestry enterprises [28]. The results would also add to the growing body of information on the role of chemotropism and sex pheromone receptors in the pathogenesis of filamentous fungal pathogens.

## 2. Materials and Methods

### 2.1. Fungal Isolates, Culture Conditions and DNA Extraction

In this study, two wild-type isolates (FSP34 and CMWF1218) of *F. circinatum* and their six corresponding mutant strains were used (Table 1). Strain FSP34 is a MAT1-1 strain and was isolated from pitch canker-affected Monterey pine (*Pinus radiata*) in California (USA) [33,34]. CMWF1218 (also known as FCC3578) is a MAT1-2 strain and was isolated from a diseased nursery seedling of Mexican weeping pine (*P. patula*) in South Africa. The mutant strains included four *ste2* green fluorescent protein (GFP) knockout mutants. Of these, two were produced from the FSP34 strain (i.e., Δ*Fcste2-1* and Δ*Fcste2-2*) and the other two from strain CMWF1218 (i.e., Δ*Cmste2-1* and Δ*Cmste2-2*). The remaining two mutants represented complementation strains (i.e., Δ*Fcste2C* and Δ*Cmste2C*) where *ste2* was re-introduced into the genomes of the mutant strains. Additionally, for assays involving synthetic α-factor peptide pheromone (see below), two additional wild-type isolates of opposite mating-type were used (i.e., CMWF1217 and CMWF1219).

All fungi are maintained in the culture collection of the Forestry and Agricultural Biotechnology Institute (FABI), University of Pretoria, South Africa. For routine culturing, the fungi were grown for 7 days at 25 °C on potato dextrose agar (PDA; 20 g/L BD Difco^TM^ Potato Dextrose Agar; Becton, Dickinson and Company, Franklin Lakes, NJ, USA).

DNA was extracted from the fungi using two methods. The first utilized PrepMan Ultra Sample Preparation Reagent (Thermo Fisher Scientific, Waltham, MA, USA) to obtain crude DNA extracts for subsequent PCR-based screening of the putative mutants. For the preparation of high-quality genomic DNA, mycelium was taken from the actively growing margins of 7-day-old cultures and used to inoculate 100 mL of potato dextrose broth (PDB; Becton). Following incubation for seven days on an orbital shaker (Orbishake 262, Labotec, Midrand, South Africa) at 200 revolutions per minute (rpm) and room temperature, mycelia were collected by filtration through 2–3 layers of sterile Miracloth (Merck KGaA, Darmstadt, Germany). These fungal tissues were then freeze-dried and subjected to DNA extraction using CTAB (N-cetyl-N, N, N-trimethyl-ammonium bromide) as described previously [35]. Following spectrophotometric quantification (NanoDrop ND-2000, Thermo Fisher Scientific), the extracted CTAB-extracted DNA was stored at −20 °C until use.

### 2.2. Identification of ste2 Gene

The *ste2* gene sequence in the genome of *F. circinatum* FSP34 was identified using the known *ste2* gene of *F. oxysporum*, *FOXG_10633* [15]. For this purpose, the *FOXG_10633* sequence was retrieved from the database of the National Centre for Biotechnology Information (NCBI; https://www.ncbi.nlm.nih.gov) using accession number XM_018390091, while the FSP34 genome was retrieved using accession number AYJV00000000. The *FOXG_10633* sequence was then used as query in a BLASTn search of the FSP34 genome sequence using CLC Main Workbench version 8.1.2 (Qiagen Bioinformatics, Aarhus, Denmark). We then confirmed that the *ste2* gene identified in *F. circinatum* contained the expected transmembrane motifs using the SOSUI [36]. We also compared it with the known *ste2* gene from *F. graminearum* (FGSG 02655; accession number XM_011320304; [37,38]).

### 2.3. Gene Disruption and Complementation

For an efficient disruption of *ste2* from the two wild-type strains, the split-marker homologous recombination approach was employed [39,40] using the methodology developed by Phasha and colleagues [35]. The selectable markers used were obtained from plasmids SK2241 [41] and pGEN-Not-1 [42]. The relevant PCRs (see below) utilized the CTAB-extracted DNA, while the primers used for generating the respective constructs were designed using CLC Main Workbench, (see Supplementary Tables S1 and S2 for primer sequences and PCR annealing temperatures).

Split-marker constructs were generated by obtaining fragments of the regions flanking *ste2* in both wild-type strains, as well as overlapping fragments of the region flanking the GFP gene (*ZsGreen*) and the hygromycin B (*hyg)* resistance gene cassette in plasmid SK2241 (see Supplementary Figure S1). We used Phusion high-fidelity PCR Master Mix (Thermo Fisher Scientific) in reactions with primer sets RP8+RP7A and RP6A+RP5, respectively, to amplify the 5’ and 3’ flanking fragments of the *ste2* gene in both wild-type strains. The Phusion high-fidelity PCR Master Mix was also used to amplify the two overlapping *ZsGreen/hyg* fragments from plasmid SK2241 using primer sets ZS-HYG-F1+ZS-HYG-HYR and ZS-HYG-YGF+ZS-HYG-R1. By making use of LongAmp Taq 2X Master Mix (New England Biolabs, Ipswich, MA, USA) and the manufacturer’s guidelines, we then “fused” the 5’ flanking fragment of *ste2* with the first *ZsGreen*/*hyg* fragment using primer set RP8+ZS-HYG-HYR. Similarly, the 3’ flanking fragment of *ste2* was fused with the second *ZsGreen*/*hyg* fragment using primer set ZS-HYG-YGF+RP5.

The deletion constructs generated were then used to transform protoplasts of the respective wild-type strains as described previously [35,43]. This involved incubation of the respective constructs with protoplasts of the wild-type strains, and recovery of mutants based on their capacity to grow in the presence of the aminoglycoside antibiotic hygromycin B (Sigma-Aldrich, Mannheim, Germany). Putative transformants were then transferred to PDA containing 150 µg/mL hygromycin B and grown as described above.

To complement the mutant strains, the *ste2* gene was randomly integrated into the genomes of the deletion mutants using the geneticin resistance gene *nptII* from plasmid pGEN-Not-1. This was performed with the Phusion high-fidelity PCR Master Mix using primer set RP8+RP5A to amplify *ste2* from each wild-type strain, and using primer set TY22+TY23 to amplify the *nptII* gene. As before, the LongAmp Taq 2X Master Mix was used to generate the fusion construct (*ste2*-*nptII*) using primer set RP8+TY23. The same procedure was used as described above for transformation with the complementation construct, except that the protoplasts obtained from the deletion mutants were used, and resistance to Geneticin G-418 (120 µg/mL, Thermo Fisher Scientific) was used for the recovery of transformants.

Initial screening for mutant transformants was conducted using the PrepMan-extracted DNAs, PCRs with Super-Therm *Taq* DNA Polymerase (Thermo Fisher Scientific) and the reaction conditions described previously [44]. Primer set Pre2GeneF+Pre2GeneR was used to verify the absence of the *ste2* gene, while primer set ZS-HYG-YGF+ZS-HYG-HYR was used to verify the presence of *ZsGreen*/*hyg*. Pure cultures for all the confirmed mutants were then prepared by overnight incubation of spore suspensions on water agar medium and then using single germinating conidia to inoculate fresh PDA. These fungal cultures were then examined for GFP expression using fluorescence microscopy.

To confirm that the *ZsGreen*/*hyg* construct correctly replaced *ste2* in the genome, PCR reactions were conducted using CTAB-extracted DNA for the mutant and wild-type strains and the amplicons sequenced. This was performed with the LongAmp Taq 2X Master Mix in reactions with primers CFPRE2-1+CF-PRE2-2 (designed outside of the flanking gene constructs). We also sequenced each of the flanking regions and the *ZsGreen/hyg* construct, following PCR with LongAmp Taq and primer sets CF-PRE2-1+ZS-HYG-HYR and ZS-HYG-YGF+CF-PRE2-2. The two amplicons were cleaned using the Zymo DNA Clean and Concentrator Kit (Zymo Research, Orange, CA, USA) and sequenced as described previously [30] with the respective PCR primers and relevant internal primers (i.e., ZS-HYG-F1, COVER-PRE2, ZS-HYG-R1 and RP6).

Copy number of *hyg* in the genomes of the mutants was evaluated with Southern hybridization analysis [45,46] using Roche’s DIG DNA Labelling and Detection Kit and DIG Easy Hyb™ (Merck). For this purpose, primer set ZS-HYG-YGF+ZS-HYG-HYR was used to generate the 562-base pair (bp) DIG-labelled *hyg* probe, while Southern blots were prepared using 20–25 µg/µL of FastDigest *Hin*dIII and *Bam*HI (Thermo Fisher Scientific) digested CTAB-extracted DNA for each of the four mutants of the two wild-type strains. Similarly, the complementation mutants were screened by Southern blot analysis for the presence of the *ste2* gene by using a probe made with primer set Pre2Gene-F+Pre2Gene-R. PCRs for making the respective probes utilized Super-Therm *Taq* DNA Polymerase.

### 2.4. Quantitative Chemotropism Plate Assay

The chemotropic effects of six test compounds were analyzed in this study. Three of these were the nutrients glucose, glycerol, and glutamate, which typically provoke chemotropic responses in fungi and generally utilize GPCRs other than Ste2 [47]. The fourth compound tested was the α-factor peptide pheromone of *F. circinatum* that was expected to interact with Ste2 [48], while the fifth compound was horseradish peroxidase (HRP), known to elicit chemotropic responses in fungi [10,15,16,17]. These five test compounds were prepared as described previously [15], which involved the use of sterile water to prepare 50 mM glucose, 15% (*v*/*v*) glycerol and 295 mM sodium glutamate (Sigma Aldrich), 4 µM HRP (Sigma-Aldrich). For comparison, peroxidase activity was abolished by adding 60 mM sodium L-ascorbate (Sigma Aldrich) directly to the HRP solution, followed by 5 min of incubation at 95 °C for 10 min. The lyophilized peptide (WCTWRGQPCW) of the synthetically produced α-factor pheromone of *F. circinatum* (GenScript, Piscataway, NJ, USA) was dissolved in 0.1% (*v*/*v*) of dimethyl sulfoxide and then assayed at a concentration of 378 μM [15]. In all these cases, water was used as the negative control in the plate assays.

The sixth compound tested was root exudate obtained from the roots of 7-month-old Mexican weeping pine seedlings. The exudate was prepared by gently uprooting the plants from their growth substrate and carefully washing them under tap water. Following a final round of rinsing with sterilized water, the plants were placed in 500 mL Erlenmeyer flasks containing 400 mL of autoclaved distilled water, ensuring that only the roots were underwater. After covering flask openings with foil, plants were incubated for 5 days at 24 °C in a phytotron. After the removal of seedlings, the exudate-containing water was vacuum-filtered through Grade 3-HW filter membranes (Merck). Filtrates were stored in sterile 50 mL falcon tubes at −20 °C until use.

The chemotropic effects of the six test compounds were evaluated using a modified version of the plate assay (Supplementary Figure S2) reported by Turrà et al. [15]. Briefly, 7-day-old PDA cultures were flooded with 15% glycerol, and 10 μL of the conidia-containing mixture was suspended in 8 mL of 0.5% (*w*/*v*) agar (cooled to around 50 °C) to a final concentration of 2.5 × 10^6^ conidia per mL. The agar suspension was poured into a 90 mm Petri plate and left to solidify. A central scoring line was then drawn on the bottom of the plate, and two parallel wells were cut on both sides into the medium at a 10 mm distance from the central line. Then, 50 μL of the test compound solution and the control were added to the wells on either side of the central line, respectively. Following the application of the test compounds, plates were incubated at 25 °C in the dark for 14 h. For each isolate, the assay was performed ten times with a particular compound. The growing direction of conidial germ tubes was recorded using a Nikon Eclipse Ni binocular microscope (100× magnification) with an affixed Nikon Ds-Ri2 camera (Nikon, Tokyo, Japan) and associated Nikon RIS Elements software (version BR 4.30.00).

A total of 200 germinating conidia were scored for each test compound, by considering only microconidia with single germ tubes. In all cases, the chemotropic responses towards compounds were compared with a negative control plate where water was added to both the test and control wells. For each strain, we quantified responses to the different compounds by determining a chemotropic index (CI). This was performed using the formula (*H*_test_ − *H*_solv_)/*H*_total_ × 100, where *H_test_* was the number of germ tubes pointing to the test solution, H_s_ was the number of germ tubes pointing to the control and *H_total_* was the total number of germ tubes counted [15]. The data were subjected to analyses of variance (ANOVA) using GraphPad Prism version 9.4.1 (https://www.graphpad.com). Significant differences between treatments were determined at a 5% significance level, while differences between means were evaluated with Tukey’s post hoc tests.

## 3. Results

### 3.1. Identification of ste2 Gene

Sequence comparisons with *F. oxysporum FOXG_10633* allowed for the identification of a single *ste2* homologue in the genome of strain FSP34 of *F. circinatum*. Unlike the *ste2* genes of *F. oxysporum* and *F. graminearum*, the *F. circinatum* gene had one intron with a length of 61 bp. In terms of the coding region (i.e., the gene sequence without the intron), the *F. circinatum* gene shared 93% identity at an Expect-value (E) of 0.0 with the coding sequences of *ste2* in *F. oxysporum*, and 76% identity with the one in *F. graminearum* (E = 7 × 10^−149^). The predicted protein in both *F. circinatum* and *F. oxysporum* contained 380 amino acid residues, and the two polypeptides shared 94% identity. As expected, the predicted protein for the *F. circinatum* gene represented a membrane protein with seven transmembrane helices [19]. We thus regarded the gene identified in *F. circinatum* as *ste2* (NCBI accession number OR260338.1).

### 3.2. Gene Disruption and Complementation

Following the *ste2* disruption experiments, we selected eight hygromycin-B-resistant colonies as putative transformants for each of the MAT1-1 and MAT1-2 wild-type strains for further characterization. PCR screening for the absence of *ste2* and the presence of *hyg* identified five suitable transformants for each of the wild-type isolates. Southern blot analysis then allowed for the identification of mutants that had an integration of only one copy of *hyg* into the genome. From these single-copy *hyg* mutant strains, we chose two for the MAT1-1 wild type (designated ∆*Fcste2-1* and ∆*Fcste2-2*) and two for the MAT1-2 wild type (designated ∆*Cmste2-1* and ∆*Cmste2-2*) to use in subsequent experiments. Additionally, complementation of the selected deletion mutants with *ste2* resulted in only a few colonies growing on PDA amended with Geneticin. Of these, PCR screening showed that only two of the MAT1-1 mutants contained the *ste2* gene, and, of these, we selected one (designated ∆*Fcste2C*) for further experimentation. The one MAT1-2 mutant containing the *ste2* gene (designated ∆*Cmste2C*) was selected for further use.

### 3.3. Deletion of ste2 Does Not Affect Chemotropism towards Glucose, Glycerol and Glutamate

**Figure 1:**
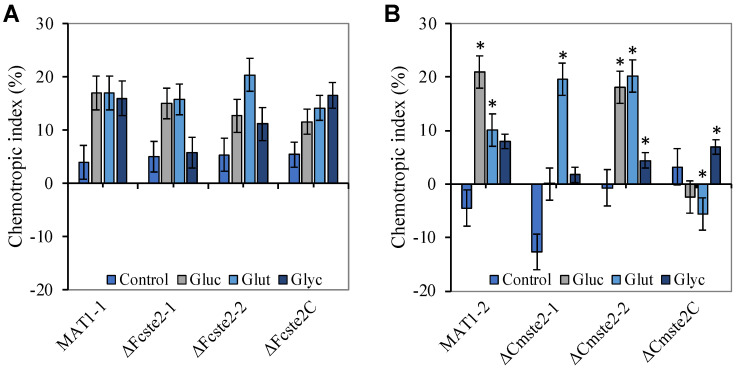
Chemotropic index values obtained for *Fusarium circinatum* in the presence of glucose (Gluc), glycerol (Glyc), and glutamate (Glut). Graphs indicate the directed hyphal growth of the MAT1-1 (**A**) and MAT1-2 (**B**) wild-type strains and their respective *ste2* deletion mutant strains (Δ*Fcste2-1* and Δ*Fcste2-2*, as well as Δ*Cmste2-1* and Δ*Cmste2-2*) and complementation mutant strains (Δ*Fcste2C* and Δ*Cmste2C*) towards the different compounds, compared to the control (sterile water) after 14 h of exposure. Means that differed significantly (*p* < 0.05) from those obtained for the control treatment are indicated with asterisks (*). Data represent the average from at least two experiments. n = 250 germ tubes. Error bars represent standard deviation.

Different chemotropic responses were elicited by the test compounds on the conidia of the two *F. circinatum* wild-type strains (Figure 1). The respective responses of the MAT1-1 wild type were not distinguishable from those evoked in the presence of water only. The chemotropic index values recorded for the glucose, glycerol and glutamate treatments were not significantly different from those observed for the control treatment with water. However, for the MAT1-2 wild-type strain, chemotropic index values recorded for the glucose and glutamate treatments differed significantly from those of the water control (*p* < 0.05).

The three test compounds also provoked different chemotropic responses from the mutant strains, as well as the complemented mutant strains of the two wild types (Figure 1A,B). For the MAT1-1 strains, neither of the mutants (∆*Fcste2-1* and ∆*Fcste2-2*), nor the complemented mutant strain (∆*Fcste2C*) provoked responses that were significantly different from that of the wild type (Figure 1A). In other words, *ste2* deletion did not affect the chemotropic responses of wild-type MAT1-1 conidia in the presence of glucose, glycerol, and glutamate.

Similarly, for the MAT1-2 strains, chemotropic index values significantly higher (*p* ≤ 0.05) than those for the wild type (Figure 1B) were recorded for the glutamate treatment of both deletion mutants and ∆*Cmste2-2* in the glucose treatment. Mutants ∆*Cmste2-1* and ∆*Cmste2C* generally lacked chemotropic response towards glucose. The MAT1-2 wild type and ∆*Cmste2* mutants did not differ significantly when exposed to glutamate and glycerol when compared to ∆*Cmste2C* and the wild type, respectively (Figure 1B). This indicated that *ste2* deletion in the MAT1-2 wild type abolished chemotropism of its conidia to glucose in one of the deletion mutants (∆*Cmste2-1*) and not the other (∆*Cmste2-2*), while the presence of the *ste2* gene did not appear to affect chemotropism in the presence of glycerol and glutamate in either of the deletion mutants.

### 3.4. Ste2 Is Required for Chemotropism towards α-Factor Pheromone

**Figure 2:**
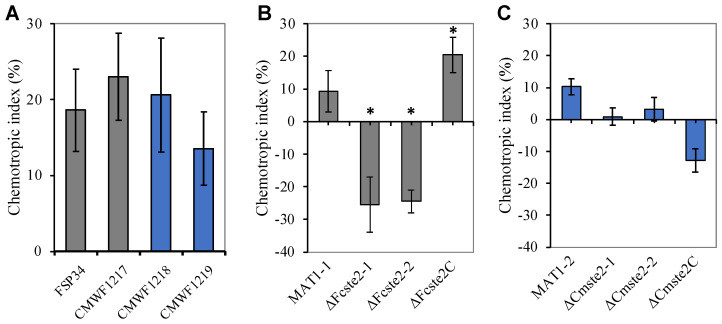
Chemotropic index values obtained for *F. circinatum* in the presence of synthetic α-pheromone peptide. (**A**) Chemotropic responses of two wild-type isolates carrying the MAT1-1 mating-type (i.e., FSP34 and CMWF1217) and two with the MAT1-2 mating-type (i.e., CMWF1218 and CMWF1219). (**B**) Chemotropic responses for the FSP34 MAT1-1 wild type and its corresponding *ste2* deletion mutants (Δ*Fcste2-1* and Δ*Fcste2-2*) and complementation mutant (Δ*Fcste2C*). (**C**) Responses for the CMWF1218 MAT1-2 wild type and its respective mutants (Δ*Cmste2-1*, Δ*Cmste2-2* and Δ*Cmste2C*). Means that differed significantly (*p* < 0.05) from those obtained from the respective wild-type strains are indicated with asterisks (*), while data represent the average of at least ten replicates. n = 250 germ tubes. Error bars represent standard deviation.

The MAT1-1 and MAT1-2 wild-type strains did not differ from each other when exposed to synthetically produced α-factor pheromone of *F. circinatum*. This finding was confirmed by examining the responses of two additional opposite mating-type strains, CMWF1217 (MAT1-1) and CMWF1219 (MAT1-2). When subjected to synthetic pheromone, these strains also displayed positive chemotropism that did not differ significantly from one another or from FSP34 and CMWF1218 (Figure 2A).

Deletion of *ste2* from MAT1-1 strain FSP34 resulted in a significant (*p* < 0.05) loss of chemotropic response to α-pheromone for both mutant strains ∆*Fcste2-1* and ∆*Fcste2-2* (Figure 2B). Its complementation mutant (∆*Fcste2C*) displayed chemotropic index values comparable to that of the wild type. For the MAT1-2 strain CMWF1218, none of the responses of the various mutants differed significantly from that of the wild type. Deletion of *ste2* from this strain did not have a significant impact on chemotropism towards the α-factor pheromone in either of the mutants, which was also the case for the relevant complementation mutant (Figure 2C).

### 3.5. Pine Root Exudate Provokes Ste2-Dependent Chemotropism

**Figure 3:**
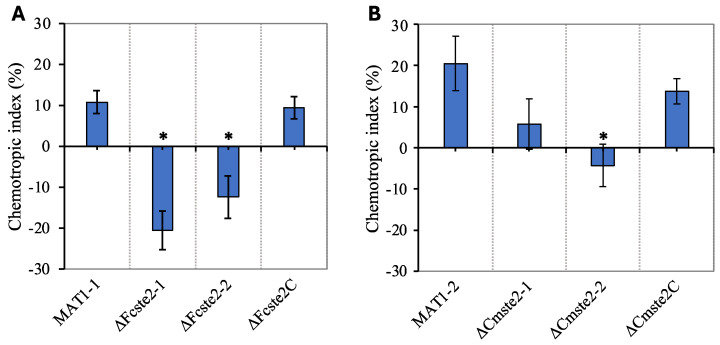
Chemotropic index values obtained for *F. circinatum* in the presence of root exudates obtained from Mexican weeping pine seedlings. (**A**) Chemotropic responses of the MAT1-1 wild type and its *ste2* deletion mutants (Δ*Fcste2-1* and Δ*Fcste2-2*) and complementation mutant Δ*Fcste2C*. (**B**) Chemotropic responses of the MAT1-2 wild-type strain and its two *ste2* deletion mutants (Δ*Cmste2-1* and Δ*Cmste2-2*) and complementation mutant (Δ*Cmste2C*). Means that differed significantly (*p* < 0.05) from those obtained from the wild-type strains are indicated with asterisks (*). Data are presented as the mean from two experiments. n = 250 germ tubes. Error bars show standard deviation.

Both wild-type strains used in this study showed positive chemotropism towards pine root exudate (Figure 3). However, deletion of *ste2* from the MAT1-1 wild type yielded significantly (*p* < 0.05) reduced chemotropic index values, although the values obtained for the corresponding complementation mutant did not differ significantly from those of the wild type (Figure 3A). Likewise, the deletion of *ste2* from the MAT1-2 wild type negatively impacted the chemotropic response towards root exudate in the two mutants, and not in the complementation mutant ∆*Cmste2C* (Figure 3B).

To evaluate whether the chemotropic responses observed might be due to the presence of peroxidases in the pine root exudate, we subjected our wild-type and mutant strains to assays using active and inactivated HRP (Supplementary Figure S3A,B). The results show that untreated HRP stimulated a positive chemotropic response in both the MAT1-1 and MAT1-2 wild-type strains of *F. circinatum,* while treatment with inactivated HRP yielded much a reduced response in the MAT1-1 wild type and a negative chemotropic response in the MAT1-2 wild type compared to when untreated HRP was used. However, these responses did not appear to be dependent on *ste2*, as responses recorded for the deletion and complementation mutants generally followed the same pattern as those observed for the wild-type strains.

## 4. Discussion

This study demonstrated that the Ste2 mating pheromone receptor of *F. circinatum* mediates positive hyphal chemotropism towards root exudate obtained from pine seedlings. In both MAT1-1 and MAT1-2 individuals, significantly (*p* < 0.05) reduced chemotropic index values were obtained when *ste2* deletion mutants were exposed to root exudates. For complementation mutants harboring a randomly integrated functional copy of *ste2*, we observed chemotropic indices comparable to those of the wild types. A similar elimination of positive chemotropism towards the roots of host plants was also observed for *ste2* deletion mutants of *F. oxysporum*, *F. graminearum*, *Verticillium dahliae* and *Trichoderma reesei* [9,10,17,24]. These results thus confirmed that Ste2 is needed for targeted hyphal growth of *F. circinatum* towards exudates released by the roots of its pine host, suggesting a primary role for the protein in this pathosystem. Accordingly, our future research will explore whether inoculation with *ste2* deletion mutants is indeed associated with reduced infection of pine seedling roots.

The precise compounds responsible for Ste2-dependent chemotropism of *F. circinatum* toward pine root exudate remains unknown, as plant-encoded class III peroxidases appeared not to be involved in the process [10,15,17,24]. These enzymes are ubiquitous in land plants [49], where they provide defense against biotic stresses by creating conditions that are toxic to potential pathogens [50]. In the current study, higher chemotropic indices were observed in the presence of HRP than in inactivated HRP and in the water control treatments for both of the *F. circinatum* wild-type strains examined, which is similar to what was seen in *F. oxysporum*, *F. graminearum*, *T. reesei* and *V. dahliae* [10,15,17,24]. However, the various *ste2* deletion mutants of *F. circinatum* yielded chemotropic index values that were not significantly different from the wild types. By contrast, those of *F. oxysporum*, *F. graminearum*, and *V. dahliae* lacked chemotropism towards HRP [10,15,17,24]. Because such a dependence on Ste2 was not seen in the *F. circinatum*, the compound(s) in pine root exudate that interacts with Ste2 thus remains to be determined. Indeed, the roots of plants, including those of pine, are known to release a diversity of compounds into the rhizosphere [4,51]. Our future research will explore the composition of the exudates for pine roots and investigate the ligand(s) potentially mediating Ste2-dependent chemotropism.

As reported for *F. oxysporum* and *V. dahliae*, the Ste2 receptor of *F. circinatum* also does not mediate chemotropic responses towards glutamate, glycerol, and glucose [10,15]. Positive chemotropic responses towards the three compounds were observed for the wild-type *F. circinatum* strains, but these mostly did not differ significantly from the control treatment nor those observed for the *ste2*-deletion mutants. This indicates that chemotropism by *F. circinatum* towards these compounds requires the activity of one or more receptor proteins other than Ste2. In *T. reesei* and *F. graminearum* GPCRs unrelated to Ste2, respectively, mediate chemotropism towards glucose [24] and nutrients extracted from wheat spikelets [52]. Investigation of the repertoire of GPCRs in *F. circinatum* might also lead to the identification of receptors mediating chemotropic responses to specific nutrients. For the latter, a suitable candidate might be strain ∆*Cmste2-1* obtained in the current study, as the generation of this mutant apparently affected target(s) in addition to the *ste2* gene, causing the fungus to behave unusually in the chemotropic assays involving glucose. This suggests that a receptor (or other relevant protein) required for chemotropism towards glucose was compromised in the mutant while retaining its chemotropic responsiveness to pine root exudates. Further examination of mutant ∆*Cmste2-1* might thus contribute to our understanding of nutrient acquisition in *F. circinatum*.

By focusing on *F. circinatum*, this study is the first to investigate Ste2-mediated chemotropism to one of the canonical pheromones in a heterothallic member of the Ascomycota. Both *F. oxysporum* and *V. dahliae* are thought to be asexual [53,54], although high levels of recent meiotic recombination were reported in a lineage of *F. oxysporum* [55]. In the case of *V. dahliae*, sexual reproduction is apparently prevented by karyotype variation that interferes with homologous chromosome pairing during meiosis [56]. Nevertheless, strains of *F. oxysporum* and *V. dahliae* harbor either the MAT1-1 or MAT1-2 versions of the MAT1 locus [57,58] and not both as is the case for homothallic species such as *F. graminearum*. The *F. oxysporum* strain in which chemotropism was studied carries the MAT1-1 version of the locus, while the *V. dahliae* strain studied carries the MAT1-2 version [15,59]. In the current study, we examined both MAT1-1 and MAT1-2 isolates in the sexual fungus *F. circinatum* and found that strains of either mating specificity responded chemotropically to synthetically produced α-peptide pheromone. Previously, synthetically produced α-pheromone for, respectively, *F. oxysporum* and *F. graminearum*, provoked high levels of positive chemotropism in these fungi [15,17], while the synthetic α-peptide from *F. oxysporum* elicited a similar response in *V. dahliae* [10]. In *F. circinatum*, deletion of the *ste2* gene also abolished the chemotropism towards the peptide, while it was restored in the complementation mutants. Therefore, under the conditions tested in the current study, our findings suggest that Ste2 of *F. circinatum* mediates positive chemotropism towards self-produced α-peptide pheromone in MAT1-1 isolates, as well as MAT1-2 isolates.

The chemotropic response to α-peptide pheromone by the *F. circinatum* MAT1-1 individual and the MAT1-2 individual contrasted with expectations for a fungus with a typical heterothallic system. We expected to observe chemotropic responses consistent with paracrine pheromone signaling (*sensu* Vitale et al. [23]) where the signals from one mating-type act on the hyphae of the opposite mating-type. This is because activation of the dedicated MAPK signaling cascade needed for sexual development requires the interaction of the α-pheromone produced by the MAT1-1 strain with Ste2 from the MAT1-2 strain, and interaction between the MAT1-2 produced **a**-pheromone and the MAT1-1 produced Ste3 [60]. Most of what we currently know about the process was gathered from gene deletion and complementation studies of model Ascomycota such as *Saccharomyces cerevisiae*, *Neurospora crassa* and *Podospora anserina*, and a small number of non-model fungi (for a comprehensive review, see [19]). Moreover, to the best of our knowledge, only the study on *T. reesei* directly examined the impact of synthetic pheromone peptides on the chemotropic responses of opposite mating-type isolates of a true heterothallic fungus [24]. However, the set of pheromones mediating mating in *T. reesei* is unusual in that its **a**-pheromone represents a hybrid between the canonical α- and **a**-pheromones. Also, *T. reesei* experiments using the synthetic **a**-peptide derived from *F. oxysporum* elicited responses resembling paracrine pheromone signaling, although assays using the *F. oxysporum* α-pheromone failed due to solubility issues [24]. Nevertheless, the collective findings of these studies highlight that there are still gaps in our understanding of sexual reproduction in Ascomycota and emphasize the importance of including non-model fungi in research enterprises.

The chemotropic responses of *F. circinatum* conidia to α-pheromone might point to one or more processes other than sexual reproduction being regulated by pheromone signaling [15,44]. The latter could include so-called autocrine pheromone signaling where the peptide signal effects changes in the cells producing it. For example, Vitale et al. [23] showed that this form of signaling in *F. oxysporum* regulates cell density-dependent germination of conidia via the CWI pathway. In this fungus, the self-produced α- and **a**-pheromones interact with the self-produced Ste2 and Ste3 GPCRs, respectively, and the corresponding α/Ste2 and **a**/Ste3 signals either promote or inhibit conidial germination via a quorum-sensing mechanism. In the MAT1-1 strain of *F. oxysporum* examined, the Bar1 aspartyl protease degrades α-pheromone at low cell densities (i.e., low α-pheromone concentrations), thus allowing for the competitive recruitment of **a**/Ste3 to the CWI MAPK cascade and the promotion of conidial germination. At higher cell densities (i.e., high α-pheromone concentrations), conidial germination is repressed because Bar1’s degradative activity is not sufficient for preventing the recruitment of α/Ste2 to the MAPK cascade. This is consistent with anecdotal observations in *F. circinatum*, where high concentrations of synthetic α-pheromone also inhibited conidial germination (unpublished). Even so, it is unclear whether a similar autocrine pheromone signaling system regulates conidial germination or other phenotypes in *F. circinatum*, but it would be interesting to explore the role(s) of α- and **a**-pheromone as quorum-sensing molecules in this fungus.

The notion that different concentrations of the various components of pheromone signaling might mediate different biological outcomes in *F. circinatum* aligns well with the limited information available for this fungus. For example, RNA-seq data on *F. circinatum* conidial germination in a MAT1-1 wild-type strain revealed nearly four times more *ste2* than *ste3* transcripts in libraries produced under similar conditions [35,61]. Additionally, the α-peptide pheromone gene of *F. circinatum* also differs widely between isolates in the number of peptide motifs included in its preprotein, as well as the exact amino acid composition of the motifs, suggesting that individuals of the fungus produce different amounts of mature peptides that vary at their N-termini [44]. The latter region in the α-peptide’s overall structure has been shown to determine growth inhibition in *F. oxysporum* [62]. Therefore, any one of these or other unknown factors could have impacted the outcome of our chemotropic assays with α-peptide pheromone and the two wild-types included, particularly in terms of the functional levels of Ste2 and α-peptide pheromone (endogenously produced and added in vitro during the assays).

## 5. Conclusions

The findings of this study shed light on our knowledge of both the pathogenesis and mating system of *F. circinatum.* Given the importance of conidia as an inoculum source for root disease outbreaks in pine seedling nurseries [63], aspects related to GPCRs such as Ste2 and the pathogen’s responses to synthetic α-peptide pheromone provide potential avenues for developing disease management strategies. In fact, Brown et al. [47] argued that the targeting of pheromone signaling for this purpose might have the dual effect of reducing a pathogen’s dispersal and its genetic diversity because of interference in its interactions with the host and with mating partners. In fact, research into how this type of information is translated into applied products is well-developed in the medical field, where pheromone signaling and GPCRs form the basis of various therapeutics for human diseases [47]. Adaptation of these proven procedures and the application of our findings might ultimately allow for the successful manipulation of the plant–pathogen interaction, thereby preventing losses due to *F. circinatum*-associated disease by pine-based industries worldwide.

## Figures and Tables

**Table 1:** List of *Fusarium circinatum* strains used in this study.

Strain	Genotype ^1^	Source ^2^
FSP34	MAT1-1 wild type	FABI
CMWF1218	MAT1-2 wild type	FABI
Δ*Fcste2-1*	Δste2: FSP34: GFP	This study
Δ*Fcste2-2*	Δste2: FSP34: GFP	This study
Δ*Cmste2-1*	Δste2: CMWF1218: GFP	This study
Δ*Cmste2-2*	Δste2: CMWF1218: GFP	This study
Δ*Fcste2C*	Δste2 + Ste2: FSP34: GFP	This study
Δ*Cmste2C*	Δste2 + Ste2: CMWF1218: GFP	This study
CMWF1219	MAT1-2 wild type	FABI
CMWF1217	MAT1-1 wild type	FABI

^1^ GFP = green fluorescence protein. ^2^ Culture collection of the Forestry and Agricultural Biotechnology Institute (FABI), University of Pretoria, South Africa.

## Data Availability

The original contributions presented in the study are included in the article/Supplementary Materials, further inquiries can be directed to the corresponding author/s.

## Supplementary Materials

The following supporting information can be downloaded at: https://www.mdpi.com/article/10.3390/pathogens13050425/s1, Figure S1: Split marker methodology used for the disruption of the *ste2* pheromone receptor gene in the wild-type genome of *F. circinatum*. Figure S2: Overview of the chemotropic assay used in this study. Figure S3: Chemotropic index values obtained for *F. circinatum* strains in the presence of untreated horse radish peroxidase (HRP) and when the enzyme was inactivated using L-ascorbate (HRP+Asc). Table S1: Primers used in this study. Table S2: PCR annealing temperatures for PCRs with the respective primer pairs used.
