# Supplementary material for: Sex Pheromone Receptor Ste2 Orchestrates Chemotropic Growth towards Pine Root Extracts in the Pitch Canker Pathogen Fusarium circinatum
Source: Pathogens. 2024 May 17;13(5):425. doi: 10.3390/pathogens13050425 (PMC11124031; doi:10.3390/pathogens13050425)
Supplement: Supplementary file 1 [file pathogens-13-00425-s001.zip › pathogens-2994883-supplementary.pdf]

Sex pheromone receptor Ste2 orchestrates chemotropic growth towards pine root extracts in the pitch canker pathogen *Fusarium circinatum*

Jane B. Ramaswe, Emma T. Steenkamp, Omotayo O. Adegeye, Felix F. Fru, Lieschen De Vos, and Brenda D. Wingfield

Supplementary Table S1. Primers used in this study.

Primer name	Primer sequence (5′-3′)
**RP-8**	AAGTTCCAGATGAAGGAGTAGG
**RP-7A**	GCATGTTGCATGATGATAAGCTTGGCACTCACTAACACTCCACAGTC
**RP-6A**	CGGCACAAAATCACCACTCGATACAAGTAGTGAAAGGAGTGAAGTGG
**RP-5**	TTAGTATTGTTCTTGCAGCTGG
**ZS-HYG-F1**	CCAAGCTTATCATCATGCAACATGC
**ZS-HYG-HYR**	ATGCAAAGTGCCGATAAACATAACG
**ZS-HYG-YGF**	GATATTGAAGGAGCATTTTTTGGGC
**ZS-HYG-R1**	TGTATCGAGTGGTGATTTTGTGCCG
**RP5A**	CAAAAATGCTCCTTCAATATCATTAGTATTGTTCTTGCAGCTGG
**TY22**	TGATATTGAAGGAGCATTTTTG
**TY23**	AGAGTAAAGAAGAGGAGCATGTC
**Pre2Gene-F**	CAAGAAGGCTTTGTCAACC
**FPre2Gene-R**	CGACCTTTGACCCGTTTAC
**CFPRE2-1**	ACCGATTTTCTCTACCCGAC
**CFPRE2-2**	ACTAGTGGCAGAGGAGAGAG
**COVER-PRE 2**	GAAACCCCCAATTTCGTCC

Supplementary Table S2. PCR annealing temperatures for PCRs with the respective primer pairs used.

Primer pair	Annealing temperature
**RP8** + **RP7A**	64°C
**RP6A** + **RP5**	64°C
**ZS-HYG-F1 + ZS-HYG-HYR**	64°C
**ZS-HYG-YGF + ZS-HYG-R1**	64°C
**RP8 + ZS-HYG-HYR**	64°C
**ZS-HYG-YGF + RP5**	64°C
**RP8-RP5A**	60°C
**TY22** + **TY23**	55°C
**RP8** +**TY23**	60°C
**Pre2GeneF** + **Pre2GeneR**	60°C
**CFPRE2-1 + CF-PRE2-2**	63°C
**CFPRE2-1 + ZS-HYG-HYR**	63°C
**ZS-HYG-YGF + CF-PRE2-2**	63°C
**ZS-HYG-YGF + ZS-HYG-R1**	60°C

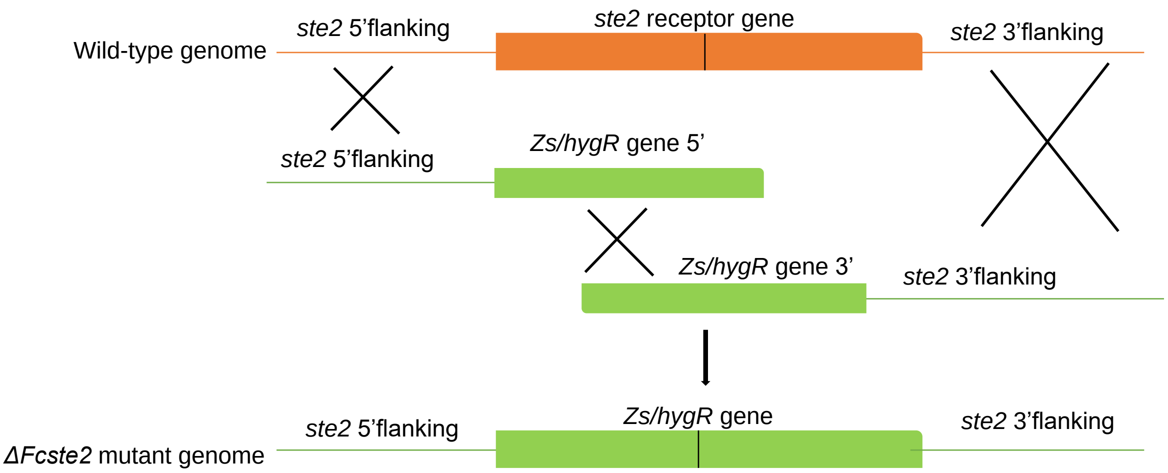

Supplementary Figure S1: Split marker methodology used for the replacement of the *ste2* pheromone receptor gene in the wild-type genome of *F. circinatum*.

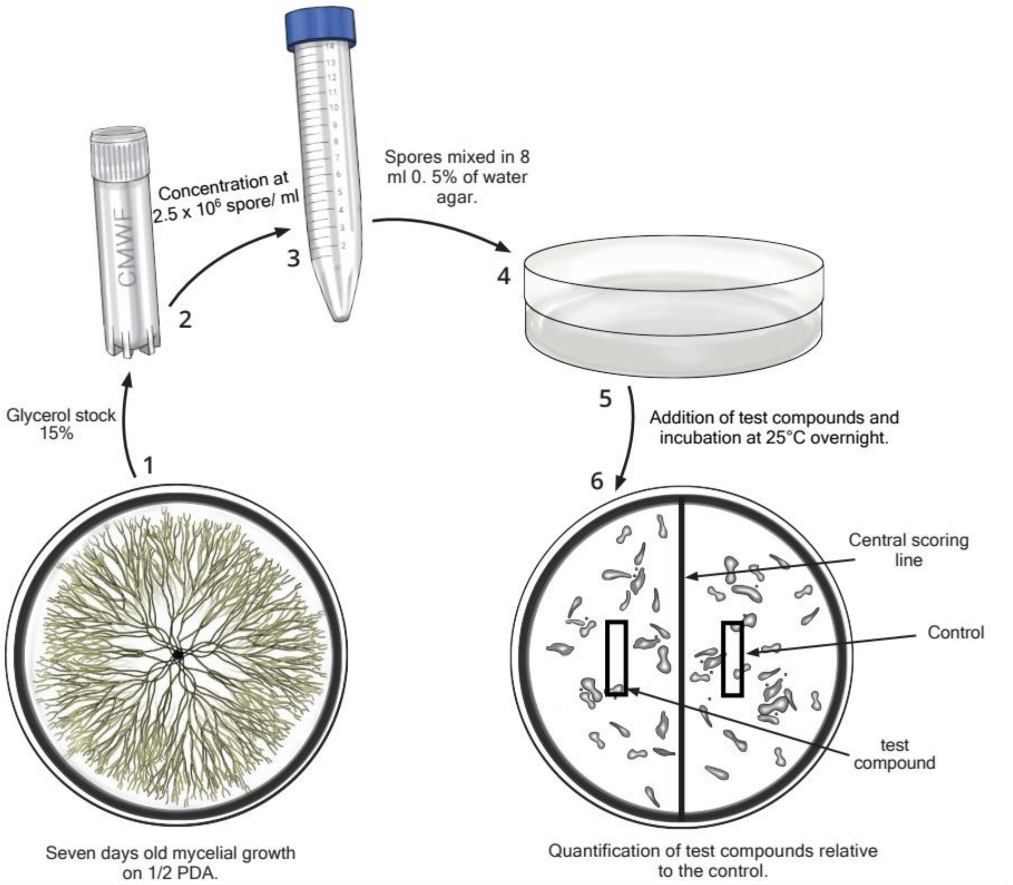

1. *Fusarium circinatum* strains were isolated on 1/2 PDA for 7 days and incubated at 25°C.
2. After incubation, spores from each strain were harvested and suspended as stock solutions in 15% glycerol and stored at - 80°C.
3. The concentration was adjusted to same to 2.5 x 10^6 spores/ml and 10 microliters of the spores was mixed with 8 ml of 0.5% water agar at 50 degrees.
4. The mixture was then poured into a 90 mm petri dish and left to solidify.
5. Fifty microliters of the control and the test compound were added into each respective wells created in the medium parallel to the central scoring line and the plates were incubated at 25 °C overnight.
6. The chemotropic effect of each test compound was quantified relative to the control by counting the hyphae of growing towards and away from the test compounds. The average was used to calculate the chemotropic index.

Supplementary Figure S2. Overview of the chemotropic assay used in this study. Solvent diffusion over the 14 hours of incubation was examined using 0.1 % (w/v) water-dissolved Congo Red (Sigma-Aldrich) as an indicator. For this 50 µL of 1% (w/v water-dissolved) Congo Red (Sigma-Aldrich) solution was added to the test well and water was added to the solvent well, and plates were incubated at 25 °C. The diffusion of the dye from the central line was recorded at different time points over 14 hours using a metric ruler, which showed a linear relationship between distance diffused and time. This showed that a solvent gradient was established over the 10 mm distance between the application well and the central scoring line.

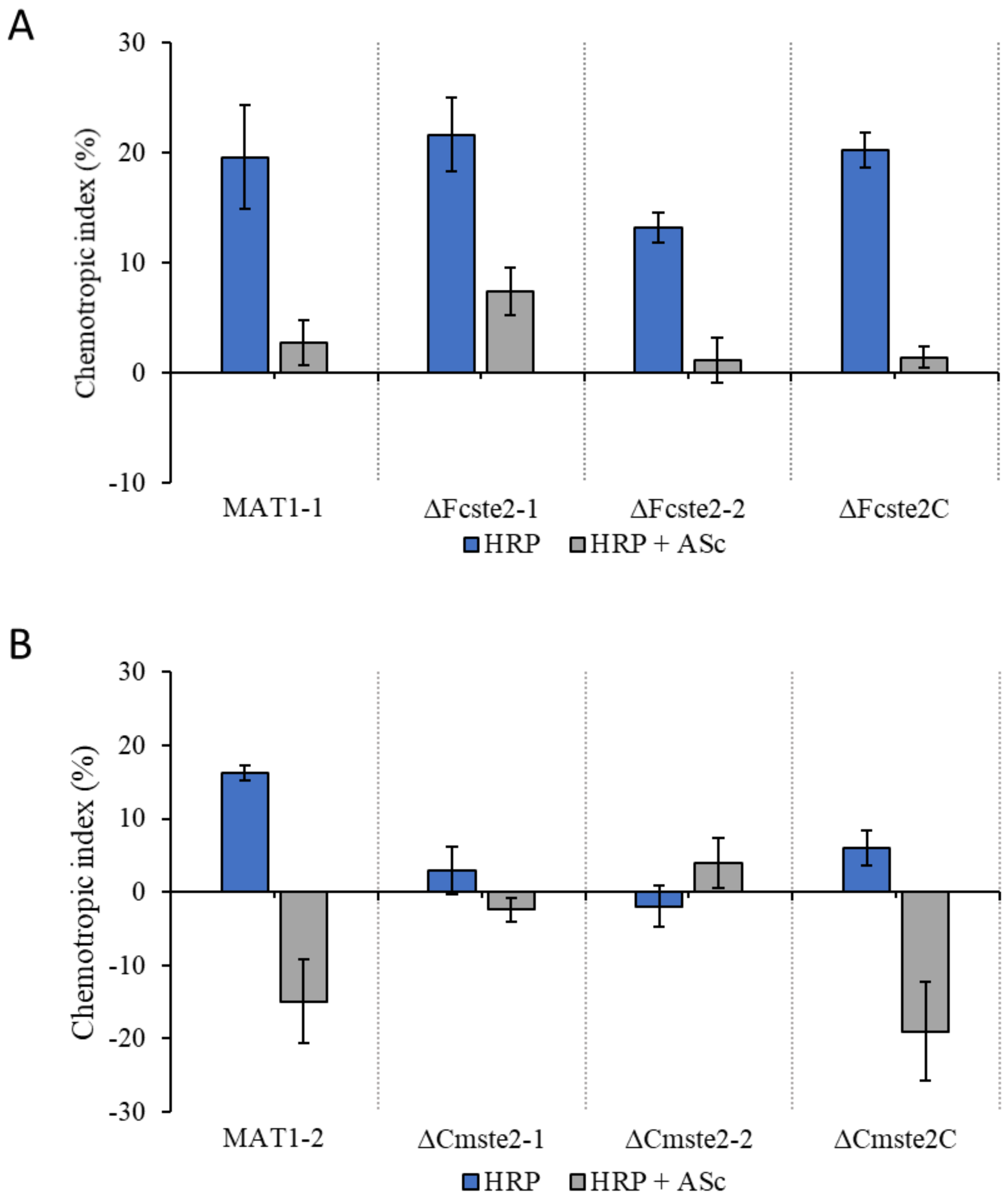

Supplementary Figure S3. Chemotropic index values obtained for *F. circinatum* strains in the presence of untreated horse radish peroxidase (HRP) and when the enzyme was inactivated using L-ascorbate (HRP+Asc). **A**: Chemotropic response of the two *F. circinatum* wild-type strains and the various deletion mutant strains and their respective complemented strains (*ΔFcste2C* and *ΔCmste2C*) towards 4 µM of HRP and HRP+ASc. Mean chemotropic index values for mutants that differ significantly ($P < 0.05$) from those of the corresponding wild-type strain are indicated with asterisks (*). Mean values were calculated from two independent experiments, each with 250 germlings scored.
